# Omics based technology application in poultry meat research

**DOI:** 10.1016/j.psj.2024.104643

**Published:** 2024-12-05

**Authors:** Huaijun Zhou, Austin Quach, Mahesh Nair, Behnam Abasht, Byungwhi Kong, Brian Bowker

**Affiliations:** a Department of Animal Science, University of California, Davis, Davis, CA, USA; b Dalton Bioanalytics Inc., Los Angeles, CA, USA; c Department of Animal Sciences, Colorado State University, Fort Collins, CO, USA; d Department of Animal and Food Sciences, University of Delaware, Newark, DE, USA; e USDA, Agricultural Research Service, U.S. National Poultry Research Center, Quality & Safety Assessment Research Unit, Athens, GA, USA

**Keywords:** Meat quality, Genetic control, Multiomics, genomics, Transcriptomics, Proteomics, Metabolomics, Lipidomics

## Abstract

Omics techniques, including genomics, transcriptomics, proteomics, metabolomics, and lipidomics, analyze entire sets of biological molecules to seek comprehensive knowledge on a particular phenotype. These approaches have been extensively utilized to identify both biomarkers and biological mechanisms for various physiological conditions in livestock and poultry. The purpose of this symposium was not only to focus on how recent omics technologies can be used to gather, integrate, and interpret data produced by various methodologies in poultry research, but also to highlight how omics and bioinformatics have increased our understanding of poultry meat quality problems and other complex traits. This Poultry Science Association symposium paper includes 5 sections that cover: 1) functional annotation of cis-regulatory elements in the genome informs genetic control of complex traits in poultry, 2) mass spectrometry for proteomics, metabolomics, and lipidomics, 3) proteomic approaches to investigate meat quality, 4) spatial transcriptomics and metabolomics studies of wooden breast disease, and 5) multiomics analyses on chicken meat quality and spaghetti meat. These topics provide insights into the molecular components that contribute to the structure, function, and dynamics of the underlying mechanisms influencing meat quality traits, including chicken breast myopathies. This information will ultimately contribute to improving the quality and composition of poultry products.

## Introduction

The suffix “-omics” refers to the comprehensive study of entire sets of biological molecules within a particular domain (Vailati-Riboni et al., 2017). For instance, "genomics" encompasses the full array of DNA within an organism, "transcriptomics" covers the complete set of RNA transcripts, "proteomics" focuses on the totality of proteins, "metabolomics" investigates the broad spectrum of metabolites, and "lipidomics" examines the entire range of lipids. By analyzing entire sets of biological molecules, these approaches help to unravel complex interactions and identify novel biomarkers or therapeutic targets, thereby shedding light on the mechanisms driving phenotypic differences (Karczewski and Snyder, 2018). Animal scientists have successfully identified specific genes, proteins, metabolites, lipids, and pathways associated with animal health and production by employing omics techniques (Dehau et al., 2022).

Advancements in animal genetics, breeding, and nutrition have led to dramatic improvements in the growth rate and efficiency related traits in modern broiler chickens (Choi et al., 2023; Zuidhof et al., 2014). However, emerging breast muscle myopathies such as wooden breast (WB), white stripping (WS), and spaghetti meat (SM) seem to be related to the increased growth rate and breast muscle yield of fast-growing broilers (Choi et al., 2023; Petracci et al., 2019). The incidence of WS and/or WB may cause $200 million and $1 billion economic loss per year in the US poultry industry (Barbut, 2020; Kuttappan et al., 2016). However, to date, the specific etiologies of muscle myopathies in broiler chickens remain unknown. It is critical to understand the specific etiologies of muscle myopathies to develop effective strategies to decrease the incidence and mitigate the impact of these myopathies on poultry production and meat quality.

Numerous studies have investigated the etiologies and functional traits of chicken breast muscle and meat quality in relation to myopathies, particularly in WB and WS (Kong et al., 2024a; Trithavisup et al., 2024; Z. Wang et al., 2023). Additionally, SM has emerged as a newly recognized muscle myopathy in broiler chickens. The advent of "multiomics," which integrates multiple types of omics data (Hasin et al., 2017), has made these investigations more comprehensive and accessible. This paper is derived from presentations in the omics symposium held at the 2024 Poultry Science Association Annual Meeting in Louisville, Kentucky to highlight the utilization of various omics technologies including genomics, transcriptomics, proteomics, metabolomics, and lipidomics in research on meat quality and other complex traits in poultry and livestock. The main three themes in this article include: 1. Genomic approaches to control complex traits in poultry; 2. Methods of mass spectrometry for proteomics, metabolomics and lipidomics; 3. Application of multiomics including proteomics, metabolomics, and lipidomics to investigate meat quality traits and chicken breast myopathies including WB and SM.

## Functional annotation of cis-regulatory elements in the genome informs genetic control of complex traits in poultry

### Huaijun Zhou, Department of Animal Science, University of California, Davis

Over the past few decades, poultry production has grown more than five-fold, making it the primary meat consumed in the United States (USDA-NASS, 2021). One of the key drivers of this growth in poultry consumption, compared to other animal products, is the tremendous advancements in poultry breeding that have resulted in commercial chickens with enhanced genetic potential. Specifically, primary breeders, who are responsible for the genetic improvements in poultry, have successfully selected birds with superior production traits and other economically important characteristics. For example, research has shown that modern broilers achieved more than 3.7-4.7 times greater body weight and are about four times more feed efficient compared to their counterparts from the 1950s, Athens Canadian Random Bred (ACRB) (Collins et al., 2014). When utilizing feeds typical for years 1957 and 2001, it was estimated that 85-90% of this improvement is attributable to genetics, with the remaining 10-15% to nutrition (Havenstein et al., 2003). The net result is greater efficiency at all levels of production, leading to a reduced environmental footprint and lower commodity prices for consumers.

Poultry remains the most affordable source of protein, and its breeding methods are now considered models for other livestock. To meet the increasing demands of consumers, the poultry industry must continue to improve selection methods in breeding programs. Similar to other farm animals, the poultry industry has adopted genomic selection (GS) to estimate the breeding value of elite individuals, which, in theory, can substantially increase the rate of genetic gain compared to traditional selection methods. A significant challenge, however, lies in identifying the underlying polymorphisms responsible for these large genetic gains. Genome-wide association studies (GWAS) have identified thousands of quantitative trait loci (QTL) or genomic regions statistically associated with phenotypic traits of interest (Ren et al., 2024). In many instances, the identified variants are not directly ‘causal’ and do not directly regulate the target gene. This occurs because, within populations, many genetic variants are co-inherited, leading to strong correlations between genotypes at different loci (linkage disequilibrium, LD) (Gallagher and Chen-Plotkin, 2018). Additional functional annotation of the putative variants is necessary to distinguish between causal and merely associated variants. Boyle et al. (2017) proposed the “omnigenic” model, suggesting that complex traits are influenced by a large number of regulatory variants of small effects active in relevant tissues. Further research has suggested that variations in complex traits are primarily driven by weak trans-eQTL (expression QTL) that regulate thousands of peripheral genes, ultimately affecting the expression of a set of core genes (Liu et al., 2019). This observation, coupled with the fact that protein-coding gene variants account for only a small proportion of the genetic variance for most complex traits, underscores the importance of identifying transcriptional regulatory elements (REs). This is further supported by evidence showing that 90% of GWAS disease-associated SNPs are located outside of coding sequences (Maurano et al., 2012). Therefore, annotating the non-coding regions of the chicken genome, particularly regulatory elements, is essential for providing functional insights into the putative genetic variants.

Following the successful Human and Mouse Encyclopedia of DNA Elements (ENCODE) projects, which functionally annotated regulatory elements in human and mouse genomes, the coordinated international effort on Functional Annotation of Animal Genomes (FAANG) has made significant strides in identifying and annotating regulatory elements in the chicken genome. Cis-regulatory elements are regions of DNA that regulate the expression of nearby genes and include promoters, enhancers, insulators. To accurately determine the positions and roles of various regulatory elements in the chicken genome, high-throughput epigenomic assays were employed. For the FAANG project, core assays include Assay for Transposase-Accessible Chromatin (ATAC-seq) for evaluating chromatin accessibility, and Chromatin Immunoprecipitation Sequencing (ChIP-seq) on the four most informative histone modifications– histone H3 lysine 4 trimethylation (H3K4me3), H3 lysine 27 trimethylation (H3K27me3), H3 lysine 27 acetylation (H3K27ac), and H3 lysine 4 monomethylation (H3K4Me1)—as well as insulator-binding protein CCCTC-binding factor (CTCF) (Kern et al., 2021). Active and inactive promoters and enhancers, along with insulators, can be characterized based on the combinations of activation marks identified in these assays (Table 1).

**Table 1:** Identification of promoter, enhancer and insulator elements based on activation marks on ATAC-seq and ChIP-seq.

Regulatory Element	Activation Marks					
	ATAC-seq	H3K4me3	H3K27me3	H3K27ac	H3K4me1	CTCF
Active Promoter	+	+	−	+	−	+/−
Inactive Promoter	+	−	+	−	+	+/−
Active Enhancer	+	+/−	+/−	+	+	+/−
Inactive Enhancer	+	+/−	+/−	−	+	+/−
Insulator	+	−	−	−	−	+

Note: + indicates peaks on the marks; − indicates no peaks on the marks; +/− indicated poised peaks on marks depends on tissues, developmental or physiological status (Cheng et al., 2021).

In the first phase of the FAANG project, a total of 23 tissues were collected from the F1 cross of two highly inbred lines: line 6 and line 7, maintained in the USDA, ARS, Avian Disease and Oncology Laboratory. These tissues were taken from two male and two female birds at the adult stage for these assays (Pan et al., 2023). An integrative hidden Markov models (HMM) (Kern et al., 2021) was applied to predict a total of 15 distinct chromatin states for each tissue. More than 1.57 million regulatory elements were identified and characterized in the chicken genome. Since enhancers are often located far from their target genes, predicting enhancer-gene pairs is crucial for elucidating underlying gene regulation. This study predicted about 1.2 million enhancer-gene pairs in the chicken genome. Furthermore, these findings were integrated with GWAS data on the economically important traits in chickens in order to pinpoint putative causal variants. The study found that GWAS linked variants were significantly enriched in biologically related tissue-specific regulatory elements. For example, growth rate-associated variants were enriched in liver, muscle, intestinal tissue-specific enhancers and promoters (Pan et al., 2023). This comprehensive atlas of regulatory elements across tissues provides a vital resource for the poultry community to study the genetic basis of complex traits and bridge the genome-phenome gap by improving genomic prediction.

While the initial landscape of regulatory elements across tissues in the chicken genome was generated, further functional annotations on context-dependent samples, such as different genetic lines, developmental stages, and physiological states, are warranted. It is well known that gene regulation acts on tissue-specific, and more importantly, cell-specific manners. Although the cost of single-cell sequencing per sample remains prohibitive for large scale assays, future functional annotation efforts should focus on at the cellular level.

## Mass spectrometry for proteomics, metabolomics, and lipidomics

### Austin Quach, Dalton Bioanalytics Inc

Mass spectrometry (MS)-based omics approaches have emerged as powerful tools for comprehensive molecular analysis in various fields, including poultry science (Cao et al., 2022; Kind et al., 2018). These techniques offer unprecedented insights into the complex biological systems that underpin avian physiology, health, and production (De Baere et al., 2012; Di Luca et al., 2024; Kind et al., 2018). This extended abstract provides an overview of proteomics, metabolomics, and lipidomics, highlighting their relevance to poultry research and potential applications.

The MS-based omics field encompasses three primary approaches: proteomics, metabolomics, and lipidomics. Proteomics is the study of all proteins within a biological system, allowing researchers to identify and quantify numerous proteins simultaneously (Aslam et al., 2017). This approach provides insights into cellular processes, signaling pathways, and structural components. Metabolomics focuses on all small molecules & metabolites present in a biological sample, offering a snapshot of the current metabolic state of an organism including exogenous and endogenous exposures such as nutrient status and utilization (Liu and Locasale, 2017). Lipidomics, the study of all lipids in a biological system, is crucial for understanding membrane biology, energy storage, and lipid-mediated signaling processes (Han, 2016).

MS provides complementary extra-genomic molecular information that is crucial for a comprehensive understanding of biological systems (Gstaiger and Aebersold, 2009). While genomics and transcriptomics have been cornerstone technologies in biological research, it's important to recognize that mRNA levels do not always correlate directly with protein levels or functional outcomes (Liu et al., 2016). MS-based omics bridges this gap by directly measuring the molecules that carry out cellular functions and transduce the phenotypes of interest (X. Wang et al., 2023).

In poultry science, these techniques have several important applications (Sidira et al., 2024). They can improve our understanding of meat quality at the molecular level by revealing changes in muscle proteins, metabolites, and lipids that affect texture, flavor, and shelf-life of poultry meat products. MS-omics can enhance animal health and welfare assessment by identifying biomarkers for various health conditions and stress responses, potentially leading to earlier interventions and improved welfare practices (Brugaletta et al., 2022; Zhang et al., 2023). These techniques can also optimize nutrition and feeding strategies by analyzing how different feed compositions affect metabolism and growth at the molecular level, guiding the development of more efficient and tailored feeding regimens (Urgessa and Woldesemayat, 2023; Zampiga et al., 2018). Additionally, MS-based techniques can address food safety concerns by detecting contaminants, residues, or pathogens in poultry products with high sensitivity and specificity (Su et al., 2024).

**Fig. 1:**
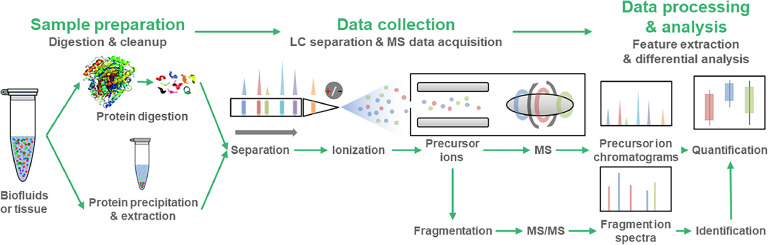
Overview of the MS-omics workflow. The process begins with sample preparation, where biological samples (biofluids or tissue) undergo either protein digestion for proteomics or protein precipitation and extraction for metabolomics/lipidomics. In the data collection phase, prepared samples undergo liquid chromatography (LC) separation followed by ionization. The resulting precursor ions enter the mass spectrometer (MS) for analysis. Ions undergo fragmentation for MS/MS analysis. Data processing involves integrating precursor intensities and fragmentation-derived identifications for quantification. Data analysis includes differential analysis to identify significant changes between sample groups.

The process of MS-omics can be broken down into three main stages: sample preparation, data collection and data processing (Fig. 1). Sample preparation involves -omics-specific sample processing. Proteomics typically involves protein extraction, cleavage of disulfide bonds by reduction and alkylation, enzymatic digestion by trypsin, and peptide sample cleanup (Su et al., 2024). Metabolomics and lipidomics often use simpler preparation procedures such as protein precipitation and extraction by solvent addition but may require different solvents or extraction techniques depending on the chemical properties of the molecules of interest, e.g. biphasic solvent or solid-phase extraction (Vuckovic, 2012).

Data collection generally involves liquid chromatography-electrospray ionization-MS (LC-ESI-MS). Liquid chromatography separates the complex mixture of analyte molecules based on their physical and chemical properties, reducing the complexity of the sample entering the mass spectrometer (Karpievitch et al., 2010). Electrospray ionization then converts the separated molecules into gas-phase ions, which are necessary for MS analysis. The mass spectrometer measures the mass-to-charge ratio (m/z) and abundance of these ionized precursor molecules, and their respective MS/MS fragmentation patterns which are subsequently used identification (De Vijlder et al., 2018). The LC-MS approaches also differ among the omics types. Proteomics typically uses long nanoflow gradients and nanospray ionization for improved sensitivity, identifying and quantifying over thousands of proteins in a single run (De Vijlder et al., 2018). Metabolomics and lipidomics usually employ shorter microflow gradients and can identify and quantify hundreds of metabolites and thousands of lipids in a single analysis (Rakusanova and Cajka, 2024).

Data processing involves quantification and identification steps. Quantification extracts the peak intensities in the MS data, with the area under each peak being proportional to the amount of the corresponding molecule in the sample (Alseekh et al., 2021). Identification matches the observed fragmentation patterns in the MS/MS spectra to known molecules using predicted or experimental spectral libraries (Kind et al., 2018). Data analysis in MS-omics generates large, complex datasets that require sophisticated bioinformatics tools. Common analytical approaches include differential analysis, co-expression module analysis, and pathway enrichment analysis. More advanced topics include the analysis of post-translational modifications in proteomics, molecular networking for improved identification of unknown metabolites in metabolomics, and detailed analysis of lipid class and acyl chain composition in lipidomics.

Each MS-omics approach provides unique insights into biological systems. Proteomics is particularly powerful for understanding biological pathways, cell type-specific protein expression, and structural composition of tissues (Xiong et al., 2024). Metabolomics excels at elucidating metabolic pathways and their regulation, as well as detecting exposures to various compounds including nutrients and environmental toxicants (Chen et al., 2023). Lipidomics provides unique insights into membrane biology, lipid-mediated signaling pathways, and cellular energetics (D. Wang et al., 2023). For an example of a meat pathology, proteomics might reveal increased abundance of extracellular matrix proteins, suggesting altered muscle structure. Metabolomics could show elevated levels of glycolytic intermediates, indicating a shift in energy metabolism. Lipidomics might identify changes in membrane phospholipid composition, potentially affecting muscle cell function. The choice of which omics to run will depend on consideration of the suspected causal mechanisms, and the options for interventions available.

While MS-omics approaches offer powerful tools for poultry science research, several challenges should be anticipated. These include the need for careful experimental design including upfront statistical power analysis, the complexity of data analysis and interpretation, and the potential for both false positives and false negatives. Collaboration with MS-omics experts and targeted follow-up validation of key findings are often necessary to extract meaningful biological insights from the data.

By understanding these principles and capabilities of MS-based omics, poultry scientists can leverage these powerful techniques to gain new insights into avian biology, improve production practices, and address key challenges in the industry.

## Proteomic approaches to investigate meat quality

### Mahesh Nair, Department of Animal Sciences, Colorado State University

The protein profile of meat influences quality attributes such as color, tenderness, and flavor. Meat color is one of the most significant attributes influencing consumer meat purchase decisions (Ramanathan et al., 2022), whereas tenderness influences consumer eating satisfaction and repurchase decisions (Shackelford et al., 2001; Troy and Kerry, 2010). Although extensively researched, some of the fundamental mechanisms contributing to meat quality differences are not completely understood. Developing a clear understanding of these processes at a biochemical and molecular level is critical in improving meat quality consistently. This section will cover different proteomic and metabolomic approaches for evaluating meat quality, both from a fresh and purified protein perspective. The focus of this section will be on beef, with the idea of developing some opportunities for cross-learning that can be applied to the poultry sector.

Previous studies have indicated muscle-specificity in beef quality attributes such as color (McKenna et al., 2005; Seyfert et al., 2006) and tenderness (Belew et al., 2003; Nair et al., 2019). The variation in postmortem metabolism among muscles, influenced by their location, physiological function, and muscle fiber characteristics, could be contributing to differences in meat quality. For example, *longissimus lumborum* (LL) and *psoas major* (PM) are two economically important muscles in beef with varying color stability. Specifically, beef LL retains its bright cherry red color for more than 6 d during retail display, whereas PM only maintains a bright cherry red color for a little longer than 24 h (Nair et al., 2018). Our initial studies using the novel tandem mass tag labeling to identify proteome changes in beef muscles during the early postmortem period indicated that greater color stability of beef LL compared to PM could be related to increased expression of anti-apoptotic proteins and the decreased expression of metabolic enzymes and proapoptotic proteins in LL (Zhai et al., 2020).

Like meat color, tenderness is also influenced by the postmortem metabolic process, specifically the activity of proteolytic enzymes during the postmortem period. Calpains (calpain-1 and calpain-2) are endogenous proteolytic enzymes present in muscles that play a crucial role in the proteolysis of cytoskeletal and myofibrillar proteins, which significantly contribute to the postmortem tenderization of meat. The muscle also undergoes severe oxidative stress during the postmortem period. The oxidation of mono- and poly-unsaturated fatty acids present in the muscle during the postmortem period can lead to the formation of various aldehydes and ketones. Previous studies have indicated that the enzyme activities in postmortem muscles could be influenced by the lipid oxidation products (Zhai et al., 2019). Therefore, we examined the effects of lipid oxidation products including hexenal, malondialdehyde (MDA), and 4-hydroxy-nonenal (HNE), on calpain-1 function and tried to identify the adduction sites. Our results indicated that calpain-1 activity was lowered by hexenal and HNE in a concentration-dependent manner, whereas there was a slight increase in activity with MDA exposure. The MDA adducts were identified on glutamine, arginine, lysine, histidine, and asparagine residues through Schiff base formation, whereas HNE adducts were found on histidine, lysine, glutamine, and asparagine residues through Michael addition. These results are the first to demonstrate that lipid peroxidation products can impact calpain-1 activity in a concentration-dependent manner and may impact the development of meat tenderness postmortem (Zhai et al., 2023).

Another meat quality attribute that significantly influences consumer eating satisfaction is flavor. Previous studies have demonstrated that postmortem aging could influence beef flavor profile (Garmyn et al., 2020). Therefore, it is important to develop technologies that can predict beef flavor. We used Rapid Evaporative Ionization Mass Spectrometry (REIMS) to evaluate whether this technology can predict the tenderness, juiciness, and flavor of beef samples after aging based on samples collected during grading (36 h postmortem). The REIMS data was paired with sensory evaluation data obtained after either 3, 14, or 28 days of aging, and different machine learning algorithms were developed to evaluate the ability of REIMS to predict eating quality. Overall, the models developed using machine learning algorithms were able to predict tenderness, juiciness, and flavor of the beef muscles with greater than 80% accuracy (Hernandez-Sintharakao et al., 2023).

In summary, proteomic and metabolomic approaches can be powerful tools for understanding changes in biological systems, especially during the postmortem period. Although the studies described above focused on beef, similar approaches could be utilized for poultry to examine color, tenderness, and flavor in finished products. In that aspect, selecting the appropriate tools/methodologies that align with the research question is crucial for obtaining the best results.

## Spatial transcriptomics and metabolomics studies of wooden breast disease

### Behnam Abasht, Department of Animal and Food Sciences, University of Delaware

Wooden Breast (WB) is a myopathy affecting modern broiler chickens, characterized by the development of stiff breast muscle tissue. This condition has significant economic implications for the poultry industry. To better understand the underlying mechanisms of WB, our studies have primarily focused on understanding its genetic basis (Lake et al., 2021) and role of lipid metabolism and oxidative stress on its onset and development (Abasht et al., 2021; Lake et al., 2019; Papah and Abasht, 2019; Z. Wang et al., 2023). We have recently used spatial transcriptomics to investigate the cellular and molecular changes associated with the early stages of WB (Wang et al., 2024). Spatial transcriptomics offers a powerful approach to analyze transcriptional patterns within distinct spatial locations of a tissue. By preserving the spatial context, this technique enhances our understanding of tissue architecture and the molecular interactions underlying histological features. While conventional transcriptomics uses whole tissue dissociation and may cause biases, spatial transcriptomics retains the regional integrity of gene expression, providing more accurate insights into the organization and function of tissues (Williams et al., 2022). Our spatial transcriptomics analysis revealed that perivascular macrophages (Papah et al., 2017), a type of immune cell, play a crucial role in the development of this condition (Wang et al., 2024). These macrophages exhibit altered lipid metabolism, with increased expression of genes involved in lipid uptake and storage. Our study also identified changes in the expression of genes related to oxidative stress, suggesting a potential link between oxidative stress and lipid metabolism in WB (Wang et al., 2024). Our prior studies (Abasht et al., 2016; Mutryn et al., 2015; Papah et al., 2018) provided further evidence for the involvement of oxidative stress and metabolic perturbations in WB. We demonstrated elevated levels of oxidative stress markers, such as cysteine-glutathione disulfide, in the breast muscles of chickens with WB (Abasht et al., 2016). Additionally, we observed alterations in glucose metabolism, with decreased glycolytic activity and increased activity of the pentose phosphate, glucosamine and glucuronic acid pathways. These findings suggest that metabolic imbalances and oxidative stress contribute to the development and progression of WB.

Taking together, our results from metabolomic and transcriptomic studies provide a more comprehensive understanding of the pathophysiology of WB. Perivascular macrophages appear to be key players in the disease process, likely contributing to the development of WB through altered lipid metabolism. The observed metabolic perturbations, including decreased glycolytic activity and increased activity of the pentose phosphate, glucosamine and glucuronic acid pathways, further highlight the complex interplay between metabolic processes and the development of this condition.

These findings have important implications for future research and interventions aimed at preventing or mitigating WB. Targeting perivascular macrophages or modulating their function could potentially be a promising therapeutic approach. Additionally, strategies to reduce oxidative stress and improve metabolic health in broiler chickens may also be beneficial. Further research is needed to elucidate the specific mechanisms underlying these processes and to develop effective interventions to address this significant health challenge in the poultry industry.

## Multiomics analyses on chicken meat quality and spaghetti meat

### Byungwhi Kong and Brian Bowker, USDA Agricultural Research Service, Athens, GA

**Fig. 2:**
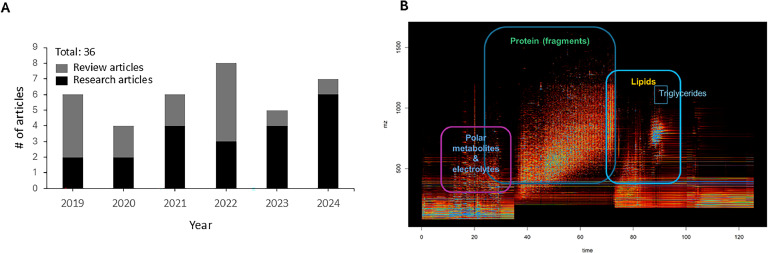
A) Number of research and review articles related to spaghetti meat. B) Example of spectrogram produced by LC-MS (provided by Austin Quach, Dalton Bioanalytics Inc.).

Spaghetti meat (SM) is an emerging chicken breast myopathy characterized by distinctive macroscopic features displaying impaired muscle integrity leading to a stringy, soft consistency on the ventral-cranial portion of the muscle. The incidence rate of SM could be up to 20% (Baldi et al., 2021). Due to its recentness, few studies have been conducted on SM (Fig. 2A), and etiologies and quality characteristics of SM are not fully understood.

While SM shares histological similarities with white striping (WS) and wooden breast (WB) myopathies, SM uniquely exhibits a progressive rarefaction of the endomysium and perimysium, along with the deposition of loose, immature connective tissue surrounding thin and split fibers (Sanden et al., 2021). These alterations in connective tissue may lead to compromised muscle integrity. According to our previous study Tasoniero et al. (2020), the SM myopathy compromised meat composition by decreasing protein content and impaired functionality traits such as water-holding capacity and emulsifying properties. Diverse omics analyses could provide valuable insights into the etiologies and functional traits of SM, offering potential strategies to: 1) reduce the incidence and severity of SM, and 2) mitigate the negative impact on its functional traits. However, to date, only a limited number of omics studies have been published, which have begun to shed light on the underlying etiologies and functional characteristics of SM.

In a previously published transcriptomics study (Che et al., 2024), SM, WB, and normal breast meat collected at 3 h post-slaughter were compared. SM and WB had 4018 and 2323 differentially expressed genes (DEGs), respectively, when compared to normal breast meat. Interestingly, there were no DEGs when SM directly compared to WB. There were 2111 shared DEGs between the comparisons (SM vs. N and WB vs. WB), suggesting that SM may retain similar physiological alterations to WB meat. Of DEGs, fifteen and thirteen collagen related genes, which directly related to formation of connective tissues, were upregulated in SM and WB, respectively. Sanden et al. (2021) demonstrated that SM may have more immature collagen fibers compared to WB. Taken together, these results suggest that SM may have upregulated collagen genes, but the immaturity of the collagen fibers may induce the detachment of muscle fibers in SM. Che et al. (2024) reported that enriched pathways with DEGs showed that DEGs were related to extracellular environment, immune response, cytokine–cytokine receptor interaction, and matrix–receptor interaction pathways in both SM and WB. SM and WB exhibit shared transcriptomic profiles; however, differences in the expression of certain genes may drive the progression towards either SM or WB.

A recent study by Wu et al. (2024) utilized LC-MS/MS to conduct a multiomics analysis of SM. There were 35 differentially abundant metabolites in SM compared to normal breast meat. The top differential metabolites included 14,15-DiHETrE, isotretinoin, L-malic acid, and acetylcysteine. These metabolites were enriched in lipid metabolism and inflammatory pathways, such as those involving linoleic acid, arachidonic acid, phenylalanine, and histidine. Our group recently conducted a metabolomics study on SM and WB at 24 hours postmortem to identify key differential metabolites in these conditions during the post-rigor mortis phase compared to normal breast meat (Choi et al., 2024). A total of 3,090 metabolites were identified in the chicken breast meat and of those, 617 differential metabolites were identified between SM and normal meat. Of differential metabolites, 15-hydroxyeicosatetraenoic acid (15-HETE) increased, and D-inositol-4-phosphate decreased in both SM and WB, while the abundance of NAD+ hydrogen (H) (NADH) was exclusively decreased in SM compared to normal breast meat. Steroid hormone biosynthesis was downregulated in SM compared to normal breast meat. This study reveals changes in both shared and unique metabolites in SM and WB, suggesting both similarities and differences in their underlying etiologies and functional traits.

**Fig. 3:**
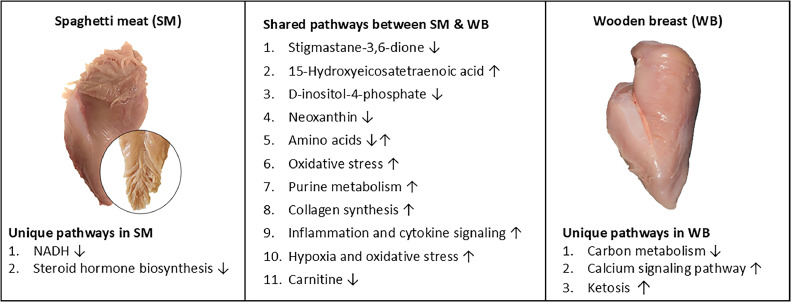
Diagrams of differential molecules and pathways between spaghetti meat (SM) and wooden breast (WB) identified by multi-omics analyses (Choi et al., 2024; Shakeri et al., 2024). Images were adapted from earlier publications (Baldi et al., 2021; Welter et al., 2022).

To date, there are no proteomic studies on SM. To understand biochemical alterations occurring in SM, our research group conducted multiomics analyses including a LC-MS assay for proteomics, metabolomics, and lipidomics (Kong et al., 2024b), which were assessed by a single MS assay (Fig. 2B) as described elsewhere in this article. A total of 2593 molecules were identified and composed of 1903 proteins, 506 lipids, 181 compounds and 3 electrolytes. There were 632 differential molecules based on *P* < 0.05 composed of 503 proteins (265 up and 367 down), 76 lipids (25 up and 51 down), 50 metabolites (14 up and 36 down), and 3 electrolytes (1 up and 2 down). Calponin was the most upregulated (fold change = 4.3) differential protein in SM compared to the normal breast meat. Pathway enrichment assay showed that differential proteins were related to carbon metabolism, glycolysis/glucogenesis, pentose phosphate pathways, etc. These pathways were frequently enriched in WB by using differential genes, proteins, and metabolomics in omics studies (Abasht et al., 2019; Choi et al., 2024; Z. Wang et al., 2023). Therefore, proteomics results suggest that SM may share similar etiologies and functional traits with WB. In addition, NAD+, lactic acid, and carnitines were decreased, while triglycerides, spermidine, and taurine were increased in SM compared to normal breast meat (Fig. 3).

In summary, results from omics studies including transcriptomics, proteomics, metabolomics, and lipidomics showed that SM exhibited both similarities and dissimilarities in its underlying etiologies and functional traits compared to other myopathic phenotypes, such as WB. Identified molecules will become mechanistic targets to reduce the incidence of breast myopathies.

## Conclusion

Multiomics studies provide valuable insights into potential genome-wide, transcriptional, and biochemical alterations in complex traits. The comprehensive knowledge derived from omics approaches can potentially be applied to develop genetic selection and nutritional intervention strategies to improve performance and product quality. To further the discoveries made possible by using multiomics approaches in meat research, the authors suggest: 1) continued refinement of methodologies will be needed; 2) methodologies to integrate individual results into a single mechanistic platform are critical; and 3) spatial approaches using vertical- or horizontal areas of tissues or single cells could provide further insight.

## Disclosures

The authors declare that they have no known competing financial interests or personal relationships that could have appeared to influence the work reported in this paper.
